# Advances in methods for colour marking of mosquitoes

**DOI:** 10.1186/1756-3305-6-200

**Published:** 2013-07-08

**Authors:** Niels O Verhulst, Jeanine ACM Loonen, Willem Takken

**Affiliations:** 1 Laboratory of Entomology, Wageningen University and Research Centre, P.O. Box 8031, Wageningen, 6700 EH, The Netherlands

**Keywords:** *Anopheles gambiae*, Marking, Survival, Host-seeking, Olfactometer, Fluorescent paint

## Abstract

**Background:**

Different techniques are available for colour marking insects and each technique may be suitable for different insect species. Mosquitoes can be marked to determine population size, distribution and flight distance or distinguish closely related species. In this study, two methods of colour marking mosquitoes were described in detail and the impact of both methods on the survival and host-seeking behaviour of the malaria mosquito *Anopheles gambiae sensu stricto* was investigated.

**Methods:**

Mosquitoes were marked in groups with fluorescent powder or fluorescent dye. The powder was applied by creating a cloud of powder in a paper cup and the dye was applied with an airbrush. The effect of marking on the survival of mosquitoes of different age groups was tested under controlled conditions. The effect of marking on the host seeking response of the mosquitoes was tested in an olfactometer with human and cow odour as baits.

**Results:**

No effect of either of the marking methods was found on the survival of mosquitoes that were treated 1 or 3 days after emergence, however, the survival of mosquitoes treated 5 or 9 days after emergence was significantly reduced. The host-seeking response of mosquitoes to human or cow odour was tested in a dual-port olfactometer and was not found to be affected by treatment with fluorescent powder or dye.

**Conclusions:**

Both methods are suitable for colour marking large groups of mosquitoes. Marking with fluorescent powder, however, is preferred because the method is simpler, visible without a UV light and no specific materials are required.

## Background

Insects are often marked to study key components of their ecology. For example, insects are marked, released and recaptured to study their population density or longevity in the field. Numerous methods are available for marking insects including: tags, mutilation, dye, dust, isotopes, genetic, radioactive-isotope and protein marking [1]. The method used should have limited or no effect on the marked insect, be cost effective, long-lasting and easy to apply. Because marking techniques can have different effects depending on which insect it is used for, preliminary studies should be carried out before conducting any investigation in which a marker is going to be used [1].

Mosquitoes have been marked to study their distribution and population density [2-5] or distinguish closely related species [6,7]. Knowledge on population size, distribution and flight distance of mosquitoes is essential when trying to reduce or eliminate mosquitoes from a certain area or estimate the force of transmission of a mosquito-borne disease. Closely related mosquito species may be difficult to distinguish, and marking them may facilitate experiments in which these species are studied together. The malaria vectors *Anopheles gambiae sensu stricto* (henceforth termed *A. gambiae*) and *A. arabiensis* for example are morphologically indistinguishable, however, they vary in their behaviour and ecology. One of the main differences is their host preference. *Anopheles gambiae* is highly anthropophilic and *A. arabiensis* has a wider host range and is considered opportunistic [8]. These differences influence their vector competence and are therefore interesting for various studies. Marking these two species differently facilitates experimental setups in which both species are used at the same time and prevents the need for PCR analysis of each individual mosquito.

The application of fluorescent dyes or powders are two techniques that are suitable for marking mosquitoes because they can easily be applied to large groups of flying insects. These dyes and powders have been used to mark many different insects, including mosquitoes [6,9,10]. Although no effect of these dyes and powders on insect performance was found in some studies [11-14], others have found a reduced insect longevity or behavioural response [15-19]. The potential negative effects of fluorescent powder and dye on mosquitoes are unknown. In this study, the impact of both marking techniques on the survival and behaviour of the malaria mosquito *A. gambiae* was investigated.

## Methods

### Mosquitoes

The *Anopheles gambiae* Giles *sensu stricto* colony originated from Suakoko, Liberia in 1988. Mosquitoes were fed on human blood obtained from the blood bank (Sanquin Blood Supply Foundation, Nijmegen, The Netherlands). The human blood was offered through a Parafilm® membrane using a Hemotek® PS5 (Discovery Workshops, UK) feeder at 38°C [20]. A sock with human odour was wrapped around the membrane and 5% CO_2_ was added to mimic a human host. Mosquitoes were kept in 30 × 30 × 30 cm gauze cages at 27 ± 1°C and 80 ± 5% relative humidity, with a 12 h light- and 12 h dark period. In the gauze cages there was a 6% glucose solution on filter paper available for the mosquitoes to feed on.

Mosquito larvae were reared in plastic trays filled with tap water and fed daily with Tetramin baby® fish food. Pupae were collected daily and placed in adult cages for emergence [21].

### Fluorescent powder

Before application of the powder mosquitoes were transferred to a 180 ml paper cup covered with gauze and anaesthetized by CO_2_ for four seconds. The cup was placed in a plastic bag just before application of the powder to prevent contamination of other materials. Pink fluorescent powder (Astral Pink, FTX Series, Swada London) was applied by filling a syringe (5 ml with 0.6 × 25 mm needle, Terumo, Leuven, Belgium) with fluorescent powder up to 0.5 ml (Additional file 1: Video 1). The syringe was held through the gauze at the top of the cup and in one gentle push the powder was blown out of the syringe. This created a cloud of powder inside the cup with the mosquitoes (Additional file 1: Video 1). Next, mosquitoes were placed on a cooling element and transferred to another paper cup (Experiment 1) or release cage (Experiment 2) using an aspirator with HEPA filter (John W. Hock, Gainesville, USA).

### Fluorescent dye

Before colouring, mosquitoes were transferred to a 180 ml paper cup covered with gauze and anaesthetized by CO_2_ for four seconds. Next, the mosquitoes were coloured with a 1:10 dilution (in water) of pink fluorescent dye (Fluorescent Pigment SC, The South Australian Research and Development Institute, manufactured by Topline Dye) (Additional file 2: Video 2). The dye was sprayed on the mosquitoes with an airbrush (starter kit BD-138, Pinearts, The Netherlands), connected to a compressor (HBM AS-16-3 Airbrush Compressor, HMB Machines BV, The Netherlands). The airbrush was held at 8–10 cm from the top of the paper cup, while rotating the cup so every mosquito was coloured (Additional file 2: Video 2). Mosquitoes were coloured till large drops were formed at the bottom of the cup (approximately 10 s). Next, mosquitoes were placed on a cooling element and transferred to another paper cup (Experiment 1) or release cage (Experiment 2) using an aspirator with HEPA filter (John W. Hock, Gainesville, USA).

### Experiment 1: Effect of marking on mosquito survival

Four different treatments were tested: control, water, dye and powder. For the control treatment the mosquitoes were anesthetised by CO_2_ for four seconds without any further treatment. The mosquitoes for the water treatment were anesthetised by CO_2_ for four seconds and after that they were sprayed for ten seconds with water with the airbrush and transferred into a clean paper cup. Powder and dye were applied as described above. To test the effect of each treatment on the survival of mosquitoes of different age groups, mosquitoes were treated 1, 3, 5 and 9 days after they emerged from pupae into adult.

Five paper cups with five female mosquitoes were tested for each treatment and for each age group. The paper cups were placed in a climate chamber at 27.5-28.0°C, RH 70-80%. Each paper cup was supplied with a piece of cotton wool moistened with 6% glucose solution. Every day the number of living mosquitoes was counted and fresh glucose solution added to the cotton wool. The cotton wool was refreshed at least once a week. Survival was recorded until all mosquitoes had died.

### Experiment 2: Effect of marking on mosquito response to host odours

The effect of marking mosquitoes with fluorescent powder or dye on the host-seeking behaviour of *A. gambiae* was investigated in a dual-port olfactometer. The olfactometer consisted of a Perspex flight chamber of 1.60 × 0.66 × 0.43 m with two trapping devices as described before [21]. Odour baits were placed in the trapping device that contained a funnel to prevent mosquitoes form exiting the trap [22]. Conditioned, humidified air (27 ± 0.5°C and 70 ± 5% relative humidity) was passed through a charcoal filter to remove traces of organic compounds [23] and through the Perspex mosquito trapping devices into the tunnel with a speed of 0.22 ± 0.02 m/s. Five cm in front of each trapping device 5% CO_2_ was released at 225 ml/min. Mosquitoes of 5–8 d old, which had never received a blood meal, were selected 14 h before the experiment and placed in a release cage with access to tap water from damp cotton wool. In each trial, 30 female mosquitoes were released from a cage that was placed downwind, 1.60 m from the two trapping devices. After 15 min, mosquitoes that entered each of the two trapping devices were counted.

The test room and flight chamber were kept at a temperature of 27 ± 1°C and a relative humidity of 65 ± 5%. The ports of the trapping devices had a temperature of 28 ± 1°C and a relative humidity >80%. Dim light was provided by a bulb (25W) with a dimmer to simulate moonlight. Gloves were worn at all time to avoid contamination of the equipment with human odours.

The effect of marking mosquitoes with powder or dye on the response to host odours was tested in two separate experiments. In each test coloured and non-coloured mosquitoes were released together in the olfactometer and given a choice between: a) human odour and no odour b) cow odour and no odour c) no odour and no odour. Each combination was tested five times on five different days. The sequence of test odours was randomized on the same day and between days. Test stimuli were alternated between right and left ports to rule out any positional effects.

Host odours were collected on nylon socks (20 denier, HEMA, Wageningen). To collect human odours a nylon sock was worn by a male volunteer for 24 hours, as *A. gambiae* has a preference to bite around the ankles [24]. To collect cow odour, socks were tied around the hind leg of a cow just above the knee. After 24 h the socks were cut into two equal pieces and stored in glass jars at −20°C. Gloves were worn to prevent contamination.

### Statistics

Differences in survival between mosquitoes coloured with powder or dye or uncoloured mosquitoes was tested with a Kaplan-Meier survival analysis (IBM, SPSS, 19.0).

A χ^2^–test was used to analyze whether the total number of mosquitoes (i.e. sum of all replicates) that was trapped in the treatment trapping device in the olfactometer and the total number that was trapped in the control trapping device differed from a 1:1 distribution (p < 0.05).

A Generalized Linear Model (GLM, Genstat for Windows, release 13.2) was used to analyze the differences in trap entry responses between mosquitoes coloured with powder, dye or uncoloured mosquitoes. The trap entry response is defined as the number of female mosquitoes caught in both trapping devices as the percentage of mosquitoes that flew out of the release cage [25].

## Results

### Experiment 1: Effect of marking on mosquito survival

**Figure 1:**
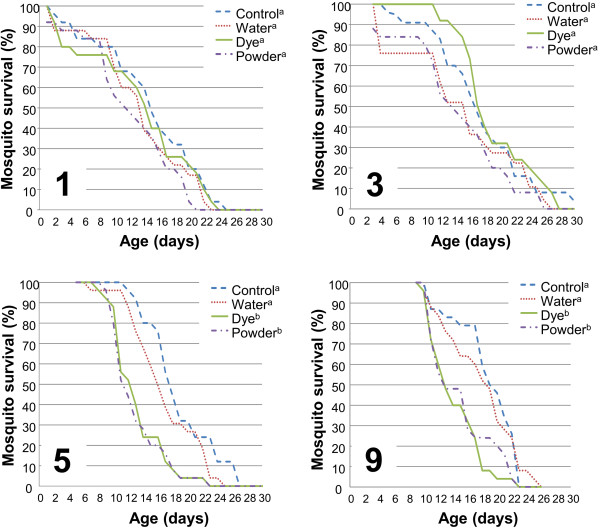
**Effect of fluorescent dye and powder on the survival of *A. gambiae*.** Mosquitoes were either non-treated (Control), sprayed with water (Water), sprayed with fluorescent dye dissolved in water (Dye) or treated with fluorescent powder (Powder). Treatments were applied 1, 3, 5 or 9 days after adults emerged from pupae. N = 25 for each treatment at each starting point. Data followed by different letters differ significantly at P < 0.05 (Kaplan-Meier).

When mosquitoes were treated 1 or 3 days after emergence, no significant difference in survival between the colouring treatments and controls were found (Figure 1, Kaplan-Meier, d.f.=1, P>0.05). Treating 5 day old mosquitoes with dye or powder significantly reduced their survival compared to the control mosquitoes (Figure 1, d.f.=1, P<0.001) and mosquitoes sprayed with water alone (Figure 1, d.f.=1, P=0.03 for dye and d.f.=1, P=0.02 for powder), indicating that the dye and powder had an effect on mosquito survival. Treatment of mosquitoes at age 5 with powder or dye lowered their mean survival to 12.32 days for dye and 12.12 days for powder compared to 17.96 for the control (Additional file 3: Table S1).

Treating 9 day old mosquitoes with dye or powder also significantly reduced their survival compared to the control mosquitoes (Figure 1, d.f.=1, P<0.01 and d.f.=1, P=0.004 respectively) and mosquitoes sprayed with water alone (Figure 1, d.f.=1, P=0.002 for dye and d.f.=1, P=0.027 for powder). Treating mosquitoes at age 9 with powder or dye lowered their mean survival to 13.40 days for dye and 14.12 days for powder compared to 18.02 for the control (Additional file 3: Table S1).

### Experiment 2: Effect of colouring on mosquito response to host odours

**Figure 2:**
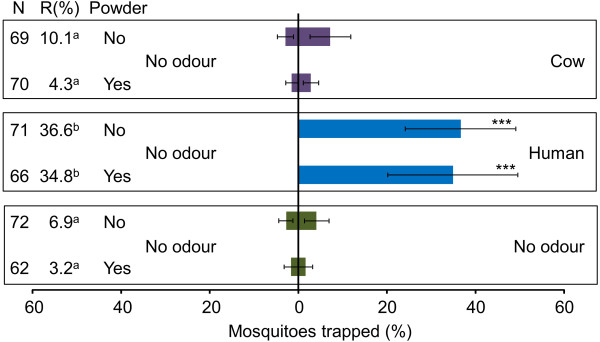
**Effect of fluorescent powder on the response of *A. gambiae *to cow and human odour.** Error bars represent standard errors of the mean; ***: χ^2^-test P < 0.001; N = number of mosquitoes released. R = The trap entry response expressed as the number of female mosquitoes caught in both trapping devices divided by the number of mosquitoes that flew out of the release cage. Data followed by different letters differ significantly at P < 0.05 (GLM).

Experiments with two unbaited traps did not show any significant differences between the left and right trapping device for both powdered and non-powdered mosquitoes (Figure 2, χ^2^=0.00, d.f.=1, P=1.000 and χ^2^=0.20, d.f.=1, P=0.655 respectively).Traps baited with human odour caught significantly more powdered and non-powdered mosquitoes than unbaited traps (Figure 2, χ^2^=23.00, d.f.=1,, P<0.001 and χ^2^=26.00, d.f.=1, P<0.001 respectively). No significant differences in mosquito catches were found between traps baited with cow odour and a control (Figure 2, χ^2^=0.33, d.f.=1, P=0.654 for powdered and χ^2^=1.29, d.f.=1, P=0.564 for non-powdered).

The trap entry response, expressed as the number of mosquitoes caught in both trapping devices divided by the number of mosquitoes that flew out of the release cage, was significantly higher during the tests with human odour (Figure 2, GLM, d.f.=40, P>0.05). Here, no differences in the trap entry response were found between powdered and non-powdered mosquitoes (Figure 2, GLM, d.f.=40, P>0.05).

**Figure 3:**
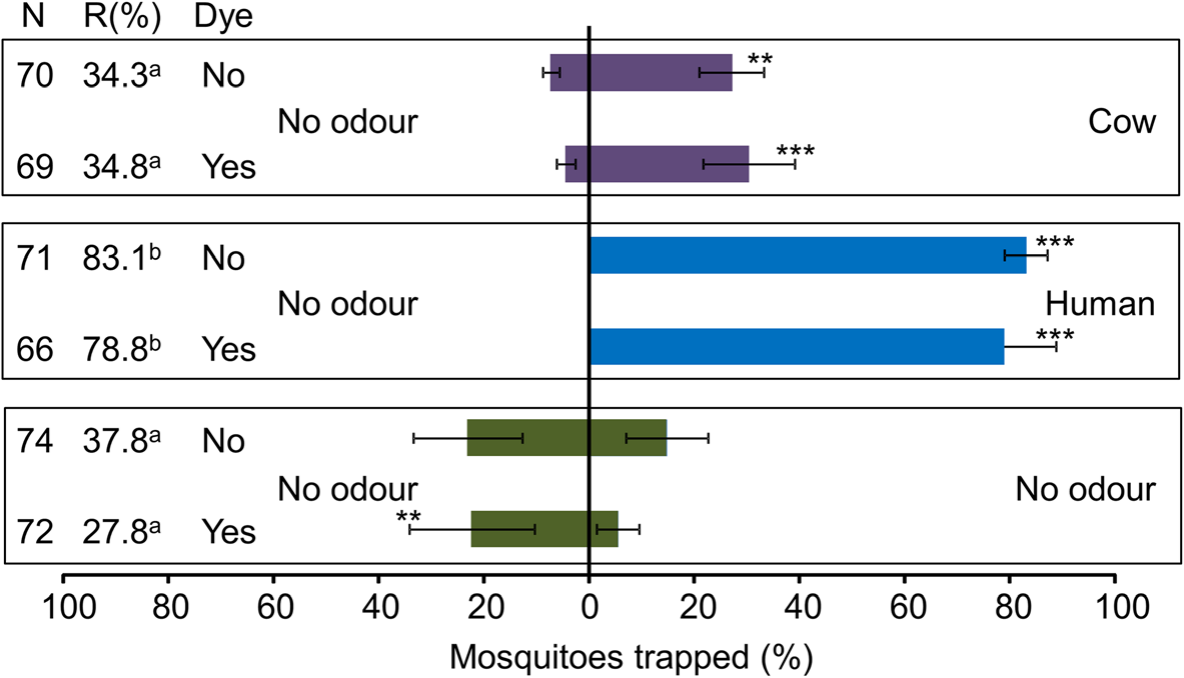
**Effect of fluorescent dye on the response of *A. gambiae *to cow and human odour.** Error bars represent standard errors of the mean; **: χ^2^-test P < 0.01,***: χ^2^-test P < 0.001; N = number of mosquitoes released. R = The trap entry response expressed as the number of female mosquitoes caught in both trapping devices divided by the number of mosquitoes that flew out of the release cage. Data followed by different letters differ significantly at P < 0.05 (GLM).

During the second series of olfactometer experiments, the overall mosquito response was higher (Figures 2 and 3). Similar to the experiments with powdered and non-powdered mosquitoes, traps baited with human odour caught significantly more dyed and non-dyed mosquitoes than unbaited traps (Figure 3, χ^2^=52.00, d.f.=1, P<0.001 and χ^2^=59.00, d.f.=1, P<0.001 respectively). Also the trap entry response was significantly higher for dyed and non-dyed mosquitoes during the tests with human odour (Figure 3, GLM including temperature effect, d.f.=39, P>0.05). Traps baited with cow odour caught significantly more mosquitoes than the control traps regardless of whether they were dyed or non-dyed (Figure 3, χ^2^=13.50, d.f.=1, P<0.001 and χ^2^=8.17, d.f.=1, P=0.004 respectively). When unbaited traps were tested, the mosquito catch in the left trapping device for dyed mosquitoes was significantly higher than the mosquito catch in the right trapping device (Figure 3, χ^2^=7.20, d.f.=1, P=0.007).

## Discussion

At the end of each survival experiment both fluorescent dye and powder could still be found on all mosquitoes tested, indicating that both methods are suitable for tracking mosquitoes throughout their life. No effect of both marking methods was found on the survival of mosquitoes that were treated 1 or 3 days after emergence. When mosquitoes were treated at an older age a significant negative effect of both colouring methods was found. No effect of the water treatment was found in any of the age groups, indicating that the longevity was reduced by the paint and dye and not by the way the mosquitoes were handled. Because marking is often used for mark-release-recapture studies, care should be taken only to mark mosquitoes that are 3 days old or younger, although results may be different in a field setting. The results of the behavioural experiments did not reveal any effect of marking on the host-seeking response of the mosquitoes. Both methods are therefore suitable for behavioural experiments in which different groups of morphologically identical mosquitoes are released simultaneously. In both behavioural experiments a high proportion of *A. gambiae* responded to human odour as was found previously [23,26]. Interestingly, in the second behavioural experiment, cow odour attracted significantly more mosquitoes than the control. This was unexpected because in previous experiments with a similar setup cow odour was not attractive or even reduced the number of mosquitoes found in trap catches when combined with carbon dioxide [23].

Studies on the effect of fluorescent powder and dye on the longevity and behavioural traits of other insects have shown various results. A negative effect of fluorescent powder on the longevity was shown for the codling moth, *Laspeyresia pomonella*[19] and cucurbit beetle, *Diabrotica speciosa*[27], but not for the mountain pine beetle, *Dendroctonus ponderosae*[12], Asian citrus psyllid, *Diaphorina citri*[18], sand fly *Lutzomyia longipalpis*[28] or the deciduous fruit moths *Aegeria pictipes* and *Argyrotaenia velutinana*[29]. Marking with fluorescent dye had no effect on the longevity of the wasp *Polistes versicolor*[30] or the parasitoid *Gonatocerus ashmeadi*[31]. In Asian citrus psyllids, *Diaphorina citri*, treated with powder, the response to a light was significantly lower up to 4 h after treatment [18] and a reduced response of powdered males to females was found in the codling moth *Laspeyresia pomonella*[19]. No effect of powder treatment, however, was found on the behavioural response of the fruit moths *Aegeria pictipes* and *Argyrotaenia velutinana*[29] and on the mobility of the sand fly *Lutzomyia longipalpis*[28]. Fluorescent dye did not influence the flight ability of fruit flies [32], but did effect grooming behaviour of the social wasp *Polistes versicolor*[30]. These studies and the study described here show that the effects of fluorescent powder or dye should be evaluated whenever using the technique for marking a new insect species.

Preliminary experiments with *Culex* mosquitoes and other *anophelines* showed that both methods are also suitable for marking groups of other mosquito species, both males and females. Additional tests should confirm what the effects of marking are on other mosquito species especially because their host preference and host seeking behaviour may be different [8]. Individual mosquitoes could be marked with the two techniques studied, however, the number of colours available is limited and ,therefore, the number of groups that can be marked is limited. Currently no reliable and easy to apply technique is available for marking individual mosquitoes or many different groups of mosquitoes. Mosquitoe larvae can be marked by feeding with stable isotopes and the mark is still detectable in adult mosquitoes [33,34]. This method, however, is more expensive than the techniques described here and requires a mass spectrometer to read the stable isotopes.

## Conclusions

An ideal marking material is durable, inexpensive, non-toxic, easily applied, and clearly identifiable [1]. Both colouring materials lasted throughout the lifespan of the mosquito with little or no effects on *A. gambiae* mosquito survival and host-seeking response. Both methods are also inexpensive, durable and non-toxic to the mosquito. However, the use of dye required specific (although cheap) equipment like an air pump and airbrush and was invisible without a UV light. Marking mosquitoes with fluorescent powder is therefore preferred, because the method is simpler and no specific materials are required.

## Competing interests

The authors declare that they have no competing interests.

## Authors’ contributions

NOV and JACML developed and executed the experimental setup and performed the statistical analysis. NOV drafted the manuscript. WT participated in the design and helped to draft the manuscript. All authors read and approved the final version of the manuscript.

## Supplementary Material

Additional file 1: Video 1 Marking mosquitoes with fluorescent powder. Click here for file

Additional file 2: Video 2 Marking mosquitoes with fluorescent dye. Click here for file

Additional file 3: Table S1 Mean and median survival times of mosquitoes treated at different ages. Click here for file
