# Supplementary material for: Advances in methods for colour marking of mosquitoes
Source: Parasit Vectors. 2013 Jul 8;6:200. doi: 10.1186/1756-3305-6-200 (PMC3708792; doi:10.1186/1756-3305-6-200)
Supplement: Additional file 3: Table S1 — Mean and median survival times of mosquitoes treated at different ages. [file 1756-3305-6-200-S3.pdf]

Additional Material Table S1

Mean and medium survival times of mosquitoes treated at different ages.

Age treated	Treatment	Mean	St. error mean	Median	St. error median
1	Control	13.80	1.31	14.00	0.99
1	Dye	12.57	1.44	14.00	0.98
1	Powder	11.28	1.17	11.00	2.49
1	Water	12.83	1.26	13.00	0.60
3	Control	14.87	1.31	14.00	1.15
3	Dye	16.34	0.97	15.00	0.60
3	Powder	13.28	1.04	14.00	1.87
3	Water	11.97	1.58	13.00	1.56
5	Control	13.96	0.88	13.00	0.82
5	Dye	8.32	0.69	8.00	1.22
5	Powder	8.12	0.69	7.00	0.55
5	Water	12.01	0.90	12.00	0.94
9	Control	10.02	0.80	11.00	1.18
9	Dye	5.40	0.69	4.00	0.99
9	Powder	6.12	0.85	4.00	1.42
9	Water	9.08	0.94	10.00	1.07
